# DNA methylation analysis of archival lymphoreticular tissues in Creutzfeldt–Jakob disease

**DOI:** 10.1007/s00401-022-02481-w

**Published:** 2022-08-18

**Authors:** Fernando Guntoro, Emmanuelle Viré, Chiara Giordani, Lee Darwent, Holger Hummerich, Jacqueline Linehan, Katy Sinka, Zane Jaunmuktane, Sebastian Brandner, John Collinge, Simon Mead

**Affiliations:** 1 MRC Prion Unit at University College London (UCL), Institute of Prion Diseases, UCL, London, W1W 7FF UK; 2 STI & HIV Department and CJD Section, Public Health England National Infection Service, 61 Colindale Avenue, London, NW9 5EQ UK; 3 Division of Neuropathology and Department of Neurodegenerative Disease, UCL Queen Square Institute of Neurology, Queen Square, London, WC1N 3BG UK

Prions are proteinaceous infectious agents of humans and animals that propagate by the templated misfolding of normal cellular prion protein (PrP) into assemblies of misfolded forms of PrP [6]. The exposure of the UK and other European populations to bovine spongiform encephalopathy (BSE) prions caused variant Creutzfeldt–Jakob Disease (CJD) and a prolonged public health crisis. Throughout, a key question has been the prevalence of vCJD prion infection in the UK population. vCJD has several distinct features including immunohistochemically detectable abnormal prion protein (PrP) in peripheral lympho-reticular system tissues (LRS, eg. tonsil, appendix). Surveys have detected abnormal PrP in the LRS of the UK population [1–5], but it remains unclear if these represent carriers of vCJD infection, some other form of prion infection, or another phenomenon altogether. Concern about the infectiousness of these possible carriers has been used to justify precautionary, expensive and ongoing health protection measures. Archival appendix samples are formalin-fixed and paraffin-embedded, that makes conventional assays of prion infection, by rodent transmission or seed amplification, challenging.

Here, we sought to use methylation array technology that assays > 850,000 sites where chemically stable DNA modification occurs to develop a computational method to classify tissue samples by prion disease status. We assembled nearly 450 lympho-reticular tissue samples from patients with different prion diseases following biopsy or autopsy, and non-prion disease patients following tonsillectomy and appendicectomy, either frozen or processed as formalin-fixed or formalin-fixed paraffin-embedded (Supplementary Table 1). In some experiments part of the frozen tissue (FR) was formalin-fixed (FF) and part of it was formalin-fixed paraffin-embedded (FFPE) to test for the effects of sample processing on DNA methylation. DNA was extracted, bisulphite converted and assayed using Illumina Infinium Methylation EPIC (850 K) BeadChips. Data were normalised and filtered, then analysed by case–control study, t-distributed stochastic neighbour embedding plots, and random forest classification methods (Supplementary Methods and Supplementary Table 2).

We first analysed tonsillar tissue derived from vCJD and sporadic CJD (sCJD) patients by tonsillar biopsy or at autopsy and from control tissue obtained at tonsillectomy. Demographic data are shown in Supplementary Table 1 and quality control (QC) data in Supplementary Table 2. Samples were necessarily imperfectly age- and sex-matched because of the distinct but overlapping profiles of patients with prion diseases and/or those having tonsillectomy or appendectomy. The purpose of this experiment was to test the hypothesis that a probable or definite prion disease diagnosis associates with a distinct profile of altered DNA methylation in lympho-reticular tissue, whilst accounting for the effects of age and sample processing. Samples that were divided and processed differently (FR, FF or FFPE) showed higher correlations than comparisons between samples from different individuals processed differently (Supplementary Table 3). A genome-wide methylation association study demonstrated substantial differences (Supplementary Figs. 1a, b) and 184 statistically significant (Bonferroni-corrected) differentially methylated sites in FR vCJD vs FR control tonsil (*P*_adj_ < 0.05, Supplementary Fig. 1, lambda = 2.69).

We used a t-SNE plot to illustrate sample similarity by two-dimensional distance based on the most variable 10% of the DNA methylation probe dataset (Supplementary Fig. 2). These plots showed evidence of higher similarity between prion disease cases than between prion disease and controls, that did not appear to be confounded by age, sex or sample processing (FR, FF, or FFPE) (see Supplementary Results). We classified tonsillar samples into vCJD, sCJD and control disease status using random forest machine learning (Supplementary Methods). 183 samples were analysed, 14 sCJD samples were classified correctly, as were 59/61 vCJD samples (errors: 1 as control, 1 as sCJD), and 89/108 controls (errors: 7 as sCJD and 12 as vCJD), with an overall accuracy of 0.885 (95% CI 0.830–0.928, *P* < 2.2 × 10^–16^, one-sided test that accuracy was better than the prevalence of the most common category (in this case, control 108/183, 59%)). Overall misclassification error score based on threefold cross-validation was 0.311 indicating a degree of overfitting in this classification.

We went onto analyse appendiceal tissues in a similar way (transparent symbols, Fig. 1). As for tonsillar tissue, t-SNE showed a clear difference between prion disease samples and controls. In the training dataset 141 samples, 72/75 sCJD samples were classified correctly (errors: 2 as control, 1 as vCJD), 16 vCJD samples were classified correctly, and 48/50 control samples (errors: 2 as sCJD) with an overall accuracy of 0.965 (95% CI 0.919–0.988, *P* < 2.2 × 10^–16^). Overall misclassification error score based on threefold cross-validation was 0.092 suggesting a lower degree of overfitting compared with the tonsillar datasets.

**Fig. 1:**
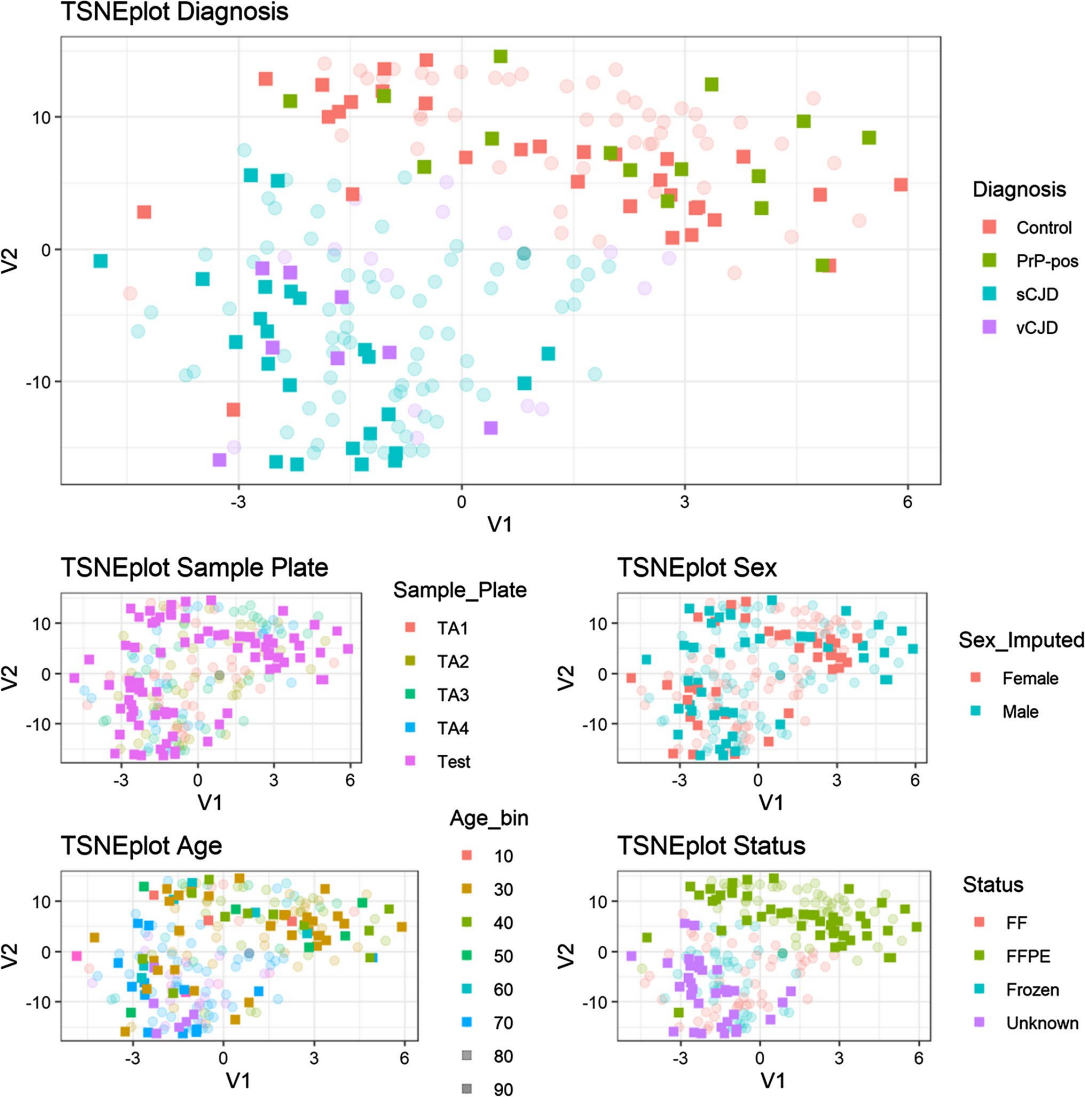
t-SNE plot of DNA methylation profiles in including training (transparent) and test (opaque) appendix samples. Five plots show the same sample locations with various overlay colours including diagnosis, sample plate, sex, age, and tissue processing status. Test samples are shown in opaque colour on top of discovery/training samples in transparent colours. Total sample size *n* = 219. PrP-pos refers to Appendix II or III samples that showed abnormal PrP immunohistochemistry

Encouraged by these findings, we processed a blinded “test” plate containing 96 samples (Supplementary Table 1, opaque symbols in Fig. 1) comprising FR and FF pairs of known prion disease appendix samples, and FFPE samples from known controls (“PrP-negative”) and those of uncertain prion disease status with abnormal PrP immunoreactivity in follicular dendritic cells of the lymphoid follicle (“PrP-positive”). t-SNE showed that prion disease cases were most similar to the prion disease cases in the training set, whereas PrP-positive and PrP-negative samples were distributed evenly in a similar distribution to known control samples. In the test dataset 78 samples, 20/22 sCJD samples were classified correctly (2 as vCJD), all 10 vCJD samples were erroneously classified as sCJD, 30/31 PrP-negative samples were classified correctly (1 as vCJD), and 14/15 test PrP-positive samples were classified as controls (1 as vCJD). The overall accuracy was 0.821 (95% CI 0.717–0.898, *P* < 1.2 × 10^–5^). A series of sensitivity analyses were conducted to assess confounding variables, tissue processing, morbidity and age (Supplementary Results).

The DNA methylation profiles of archival appendix samples with abnormal PrP were most similar to, and classified with, control appendix samples, rather than prion disease samples. Several interpretations are compatible. Whilst one might conclude that these data provide evidence against prion infection of archival appendix tissues, we think this would be an over-interpretation. Subclinical or preclinical infection may associate with poorly developed DNA methylation profiles, and confounding factors like age, inflammation in tissue, disease morbidity or tissue processing may have obscured a diagnostic profile, despite our best efforts to account for these possibilities. A fuller discussion of public health policy implications is given in Supplementary Materials.

## Supplementary Information

Below is the link to the electronic supplementary material.

Supplementary file1 (DOCX 782 KB)

## Data Availability

All data analysed in this report will be deposited on GEO (https://www.ncbi.nlm.nih.gov/geo/).
