# Supplementary material for: DNA methylation analysis of archival lymphoreticular tissues in Creutzfeldt–Jakob disease
Source: Acta Neuropathol. 2022 Aug 18;144(4):785–7. doi: 10.1007/s00401-022-02481-w (PMC9468073; doi:10.1007/s00401-022-02481-w)
Supplement: Supplementary file 1 — Supplementary file1 (DOCX 782 KB) [file 401_2022_2481_MOESM1_ESM.docx]

**Supplementary Material**

**DNA methylation analysis of archival lymphoreticular tissues in Creutzfeldt-Jakob disease**

Fernando Guntoro^1*^, Emmanuelle Viré^1*^, Chiara Giordani^1^, Lee Darwent^1^, Holger Hummerich^1^, Jacqueline Linehan^1^, Katy Sinka^2^, Zane Jaunmuktane^3^, Sebastian Brandner^3^, John Collinge^1^, Simon Mead^1**^

^1^MRC Prion Unit at University College London (UCL), Institute of Prion Diseases, UCL, London W1W 7FF

^2^STI & HIV Department and CJD Section, Public Health England National Infection Service, 61 Colindale Avenue, London NW9 5EQ

^3^Division of Neuropathology and Department of Neurodegenerative Disease, UCL Queen square Institute of Neurology, Queen Square, London WC1N 3BG

*These authors contributed equally

**Corresponding author [s.mead@prion.ucl.ac.uk](mailto:s.mead@prion.ucl.ac.uk)

Contents

[Supplementary Methods 3](#_Toc108182065)

[Supplementary Results 5](#_Toc108182066)

[Supplementary Discussion 6](#_Toc108182067)

[Supplementary Figure 1a. Manhattan plot vCJD tonsil vs case-control study. 8](#_Toc108182068)

[Supplementary Figure 1b. QQplot from the vCJD tonsil case-control study 8](#_Toc108182069)

[Supplementary Figure 2. tSNE plots of DNA methylation profiles derived from tonsillar tissue samples 9](#_Toc108182070)

[Supplementary Figure 3. tSNE plots of DNA methylation profiles derived from tonsillar tissue samples (including patient disease severity) 10](#_Toc108182071)

[Supplementary Figure 4. tSNE plots of DNA methylation profiles derived from tonsillar tissue samples 11](#_Toc108182072)

[Supplementary Table 1. Characteristics of the samples used in the study 12](#_Toc108182073)

[Supplementary Table 2. Description of array processing and quality control of the data for further analyses 13](#_Toc108182074)

[Supplementary Table 3. Correlation between paired frozen (Fr), formalin-fixed (FF) and FFPE samples, compared with unpaired samples and background correlation in each diagnosis group 14](#_Toc108182075)

# Supplementary Methods

*Samples, ethics, consent*

Appendiceal and tonsillar tissues from patients with prion diseases and controls were archived at the MRC Prion Unit at UCL and obtained with informed consent for research studies. Frozen appendiceal tissue from prion disease patients was obtained at autopsy, whereas formalin fixed and paraffin embedded control appendiceal tissues were derived from those stored following the Appendix studies. Tonsillar tissue was obtained by biopsy or autopsy (in patients) or tonsillectomy (controls). Patients with sporadic and variant CJD were diagnosed using contemporary diagnostic criteria. Genomic research studies were approved by the Queen Square local Research Ethics Committee.

In some experiments part of the frozen tissue (FR) was formalin-fixed (FF) and part of it was formalin-fixed paraffin-embedded (FFPE) to test for the effects of sample processing on DNA methylation (FR, FF or FFPE referred to as tissue fixation ‘status’ hereafter). Formalin fixation was done according to standard procedures at the MRC Prion Unit at UCL by fixation in 10% buffered formalin for 72h. After formalin-fixation, samples were dehydrated in graded alcohols and then embedded in paraffin. Because of health and safety measures at the laboratories of the MRC Prion Unit at UCL, the FFPE processing was only done for control and not prion infected tissue.

*DNA extraction from appendix and tonsillar tissues*

Possibly prion infected tissue was processed in a Biosafety Level 3 laboratory. Prior to DNA extraction, FR and FF samples were lysed using a Precellys Ribolyser and incubated in ATL lysis buffer (Qiagen) with proteinase-K overnight (50 μl proteinase K (from 20 mg/ml stock) Ambion) with mixing(7). For the FFPE tissues, dewaxing steps with Xylene were repeated twice and the tissue was digested overnight. DNA from these processed FR, FF and FFPE tissues was extracted with the Zymo Quick-DNA/RNA FFPE Kit according to the manufacturer’s instructions. DNA was cleaned using the Zymo Genomic DNA Clean & Concentrator. DNA concentration was determined with a Qubit Fluorometer. The quality of DNA extracted was also assessed using an Agilent TapeStation (as expected, the DNA integrity number (DIN) of FF and FFPE samples were lower than FR samples).

*Genome-wide methylation array processing*

DNA restoration for the FFPE samples, bisulfite conversion, and methylation array processing were done by UCL Genomics at the Zayed Centre for Research into Rare Disease in Children. DNA from FFPE samples was restored using the Infinium HD FFPE DNA Restore Kit. Bisulfite conversion was performed using the Zymo EZ-96 DNA Methylation-Gold Kit. DNA samples from different tissue, disease status and processing groups were distributed evenly across batches and hybridized onto the Infinium MethylationEPIC BeadChips to measure the methylation levels of >850,000 CpG sites.

*Data preprocessing*

Raw signal intensities were obtained from IDAT files using the minfi package in R (8). All samples were preprocessed using the preprocessIllumina() function implemented in minfi (8), which performs background correction and control normalization which is comparable to Genome Studio implementation. Beta values were calculated from the transformed intensities using an offset of 100 (as recommended by Illumina). The beta values were then normalized using the BMIQ method implemented in ChAMP (9). To test for possible confounding batch effects within the dataset, the singular value decomposition (SVD) analysis implemented in ChAMP was used (9). Subsequently, a correction for significant batch effects was performed by fitting univariate, linear models using the removeBatchEffect() function in the limma package (10). The tonsil dataset was corrected for the type of material used (‘status’), whereas the appendix dataset was corrected for the EPIC array slide, based on the SVD analysis. Several filtering criteria were applied using champ.filter() function in the ChAMP package (Supplementary Table 2) yielding 557,431 CpGs for further analyses (9).

*Correlation analysis*

Pairwise Pearson correlation was calculated for all appendix and tonsillar samples using all CpGs. Correlation statistics are summarised based on relevant comparisons between disease groups and sample status within appendix and tonsillar samples.

*Differential methylation analysis*

The differential methylation analysis was performed using a linear regression model with the limma package (10). This was used to identify differentially methylated probes (DMPs) between vCJD and control FR tonsils. DMPs with a Bonferroni-adjusted P-values less than 0.05 were considered significant. A Manhattan plot was generated using an in-house script and a significance threshold was drawn at Bonferroni-adjusted threshold of 8.97 x 10^-8^. Quantile-quantile (QQ) plots were generated using an adapted version of the pQQ function of the haplin package (11) and an area of 95% confidence level was shaded around the reference line. Kyoto Encyclopedia of Genes and Genomes (KEGG) pathways were analyzed using DAVID for genes with Adj. P Val. < 0.05 (12, 13).

*Unsupervised analysis*

A t-distributed stochastic neighbour embedding (t-SNE) analysis was performed by selecting 10% of the most variably methylated CpGs (n = 55,743) using the Rtsne package (14). The following parameters were used: initial_dims = 86, theta = 0, max_iter = 2500, perplexity = 30. Perplexity values of 5 and 50 were also tested but showed no great discrepancy. The plots were then overlaid with relevant metadata: plate, sex, age and status.

*Machine learning classifier development*

A published DNA methylation random forest classifier was adapted (15). The random forest algorithm is a supervised learning algorithm which relies on an ensemble of decision trees. To train the random forest classifier, the randomForest package was used (16). Feature selection to obtain the most important CpGs was done by applying the random forest algorithm to the beta values of pre-filtered 20,000 most variable CpGs (consider - all 557,431 CpGs). 10,000 trees were fitted and the selected features were ranked using the variable importance measure. The final random forest classifier was trained by fitting 10,000 trees using the beta values of the 20,000 CpGs selected during feature selection. The random forest model trained on the appendix Training dataset (see Supplementary Table 1 for samples used) was used to predict the disease group classification in the Test dataset (Supplementary Table 1) and produced a confusion matrix. The accuracy of the random forest model was evaluated by a threefold, nested cross-validation. The Training dataset was split into three equally sized parts, and in each iteration, two-thirds of the dataset were used to train a random forest classifier. The remaining one-third of the data were used for prediction using this random forest classifier.

# Supplementary Results

Sensitivity analyses were done that examined possible confounding effects. First, we considered the possibility that DNA methylation status in LRS tissue was determined by CJD disease severity in a general sense, rather than the presence of prions or abnormal PrP in the LRS tissue itself. In this circumstance we might expect that patients with more advanced disease would have more distinct DNA methylation profiles compared with controls than patients with less severe disease. We therefore labelled vCJD tonsil samples by whether they were obtained at post-mortem, or biopsy, and if at biopsy, then by the tercile of clinical duration (Supplementary Figure 3). We observed no obvious clustering of samples by disease severity.

In a second sensitivity analysis, we considered the possibility of confounding by tissue processing. We therefore did a genome-wide methylation association study comparing paired (n=38) tonsillar tissues processed as FF or FFPE, and separately comparing known vCJD appendix (n=8) to FFPE control appendix (n=36). We then repeated classification and t-SNE using a dataset that was filtered for the top ranked 50,000 DNA methylation probes in each genome-wide methylation association study. In both circumstances as above, 14/15 PrP-positive samples were classified as controls, and t-SNE showed PrP positive samples distributed amongst controls, overall accuracy remained similar (0.795-0.833), suggesting that tissue processing did not confound the classification to prion disease or control.

In a third sensitivity analysis we repeated the classification studies whilst adding age and sex as covariates. Overall accuracy in the test experiment of appendix DNA, without age and sex was 0.821 (0.717-0.898) with age and sex included was 0.808 (0.702-0.888). In the age and sex adjusted analysis 26/31 controls were classified correctly (4 as sCJD and 1 as vCJD), 16/22 sCJD were classified correctly (1 as control and 5 as vCJD) and 6/10 vCJD were classified correctly (1 as control and 3 as sCJD). In the age and sex adjusted analysis all 15 PrP positive test samples were classified as controls (vs 14/15 in the non-adjusted analysis). In the training tonsil and appendix analyses, the addition of age and sex improved accuracy of classification, but also increased the misclassification error rate. Overall, on the basis of this sensitivity analysis, we concluded that age and sex did have modest influences on classification, but not to the extent that this qualitatively changed our interpretations.

# Supplementary Discussion

In this study we used DNA methylation analysis of lymphoreticular (LRS) tissues from patients with prion diseases and controls to develop a new diagnostic classification tool. We showed reasonable levels of accuracy of the method across different types of tissue and processing (82-97%). We went on to apply the classifier to tissues derived from the Appendix prevalence surveys, which came from people who were healthy from the point of view of prion disease, but showed abnormal PrP in follicular dendritic cells. These samples were distributed amongst controls in t-SNE plots and classified (14 control /1 vCJD) as control samples by random forest machine learning. These findings have several caveats and interpretations discussed below and assessed in sensitivity analyses.

Our interpretations must be constrained for several reasons. DNA methylation profiling and classification methods have proven to be extremely accurate in the context of *large* training sample collections derived from patients with known diagnoses, typically cancer. Cancer tissues show marked abnormalities of DNA methylation (eg. ~3% CpG sites delta beta >0.15) because of the profound effects of neoplasia on the epigenetic status of the cell, whereas the case-control differences we observed in tonsil were remote from brain tissue that is most impacted by prion disease and therefore much more modest. The sample collections available for study were necessarily small due to the rarity of prion diseases, and the availability of diagnostic imaging and clinical criteria in life meaning that tonsillar tissues were sampled in only a small minority of cases of vCJD. The machine learning methods of classification and visualisations we used cannot be considered as robust, because typically thousands of samples are required before classification tools become stable(10).

We considered how effects of tissue fixation and processing might impact our results and used a DNA repair kit, despite the fact that there is good evidence for the stability of DNA methylation status in damaged DNA(17, 18). In sensitivity analyses we tested for the possibility of confounding by probes sensitive to processing methods by filtering out DNA methylation probes that we found were altered by tissue processing (when the same sample was divided and processed differently). Whilst we found no changes to the overall accuracy or classification of tissues of unknown status, we cannot exclude the possibility that the archival FFPE processing status of appendix tissue has a distinct DNA methylation profile that obscured the signature of prion infection.

A further important limitation is that we do not know the cause or timing of altered DNA methylation in peripheral lymphoreticular tissues of prion disease patients. This could relate directly to prion infection of the tissue, alternatively, DNA methylation changes might result from more non-specific factors related to the general ill-health of the patient, treatments given, poor nutritional state, hypoxia or infection around the time of death. Such non-specific effects or morbidity would not likely be present in the PrP-positive appendix samples and would therefore bias classification to control status. The Appendix study samples were most likely to have come from those with appendicitis (although obviously inflamed samples were excluded) (3), nevertheless other confounding diagnoses that led to appendicectomy might similarly have confounded the analysis. We have not been able to source lymphoreticular tissue samples from non-prion dementia patients that might be used to further explore this possibility. We addressed this in part by examining the effects of disease severity in vCJD on tonsillar DNA methylation, concluding this was not a strong effect, but these analyses do not eliminate the possibility of confounding by morbidities distant from the test tissue. In summary, our conclusions are caveated by the absence of a gold-standard training sample set of subclinical carriers of vCJD infection.

Finally, and necessarily, we were unable to fully match case and control samples by age and sex because of the profiles of patients with prion diseases are different from those having appendicectomy and tonsillectomy. We did analyses with and without adjusting for these variables, and concluded that these were significant in effects, but not to the extent that they altered the prediction for the PrP-positive appendices. Indeed, in the age and sex adjusted analysis all PrP-positive appendices were classified as controls.

Three potential explanations of the Appendix prevalence data may be considered: (i) that some or all of the “positive” appendices are “false” positive in that the PrP immunohistochemistry finding is a consequence of a prion-unrelated phenomenon, (ii) that some or all of the positive appendix results are prion-related, but these are not specific to vCJD infection, perhaps indicating a phenomenon related to other types of prion disease, or (iii) that the assumptions about the start of the BSE epidemic were incorrect, and in fact, BSE exposure prior to 1980 and after 1995 was sufficient to result in the findings of Appendix-III. Whilst we found that the test appendix tissues were indistinguishable from control tissues, which might favour interpretation (i) above, it would be an over-interpretation to conclude that the Appendix studies showed a “false-positive” result, as other interpretations of our work are valid. Our work needs to be considered together with other programmes designed to help interpretation of the Appendix studies, and of course the accruing epidemiological evidence of the absence of clinical vCJD in the UK population (zero known cases in the last five years).

Single cell DNA methylation might have been insightful but was not possible on these samples due to the early stages of the technological advancements in single cell-methylomics and also with the confounding factor of the sample infectivity hampering single cell isolation. Future work might explore a role for DNA methylation in the molecular classification of CJD brain tissues. If an appendix sample collection from non-prion dementia patients might be acquired, analysis of this might assist in the interpretation of our current findings. With respect to other methods of classification of the Appendix tissues, bioassay is clearly problematic because of the effects of fixation, but other methods to directly amplify prion seeds by PMCA, observe prions by electron microscopy, or assay prion titre by novel methods could all be pursued.

# Supplementary Figure 1a. Manhattan plot vCJD tonsil vs case-control study.

Supplementary Figure 1b. QQplot from the vCJD tonsil case-control study**,** showing the relationship between observed P value and expected P value of 557,431 CpGs, showing an inflation lambda value of 2.69.
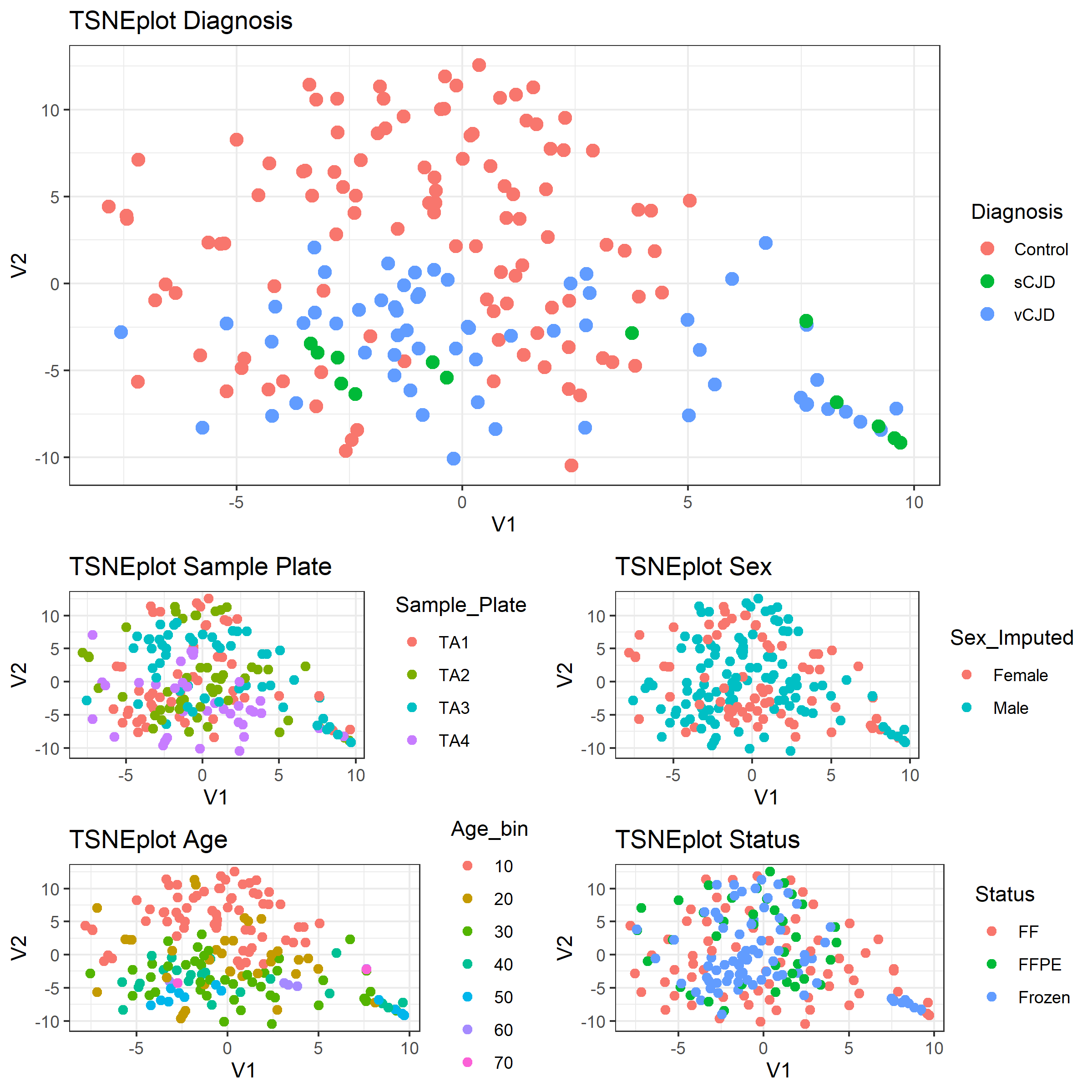


Supplementary Figure 2. tSNE plots of DNA methylation profiles derived from tonsillar tissue samples**.** t-SNE plots illustrate the estimated DNA methylation profile similarities between pairs of samples on a 2D plot. More similar profiles are shown closer together on the plots. These five plots show the same sample locations with various overlay colours including diagnosis, sample plate, sex, age, and tissue processing status. Total sample size n = 183. Duplicate samples are shown connected in Supplementary Figure 4.

Supplementary Figure 3. tSNE plots of DNA methylation profiles derived from tonsillar tissue samples (including patient disease severity). t-SNE plots illustrate the estimated DNA methylation profile similarities between pairs of samples on a 2D plot. More similar profiles are shown closer together on the plots. These five plots show the same sample locations with various overlay colours including diagnosis, sample plate, sex, age, and tissue processing status. Total sample size n = 183. For Terciles 1, 2, and 3, are the earliest, middle and late thirds of clinical duration. 4 = autopsy sample.

Supplementary Figure 4. tSNE plots of DNA methylation profiles derived from tonsillar tissue samples**.** t-SNE plots illustrate the estimated DNA methylation profile similarities between pairs of samples on a 2D plot. More similar profiles are shown closer together on the plots. These five plots show the same sample locations with various overlay colours including diagnosis, sample plate, sex, age, and tissue processing status. Total sample size n = 183. Duplicate samples are shown connected.

| Tissue | Diagnosis | Fixation status | n | Mean (SD) Age | %Female |
| --- | --- | --- | --- | --- | --- |
| *Training Dataset* |  |  |  |  |  |
| Appendix | vCJD | Frozen | 10 | 31.5 (7.2) | 20.0% |
| (Total 155) |  | Formalin-Fixed | 8* | 29.5 (6.6) | 25.0% |
|  |  | FFPE | - | - | - |
|  | sCJD | Frozen | 37 | 67.1 (9.5) | 51.3% |
|  |  | Formalin-Fixed | 38* | 67.1 (9.4) | 52.6% |
|  |  | FFPE | - | - | - |
|  | Control | Frozen | - | - | - |
|  |  | Formalin-Fixed | - | - | - |
|  |  | FFPE | 62 | 31.9 (12.6) | 41.9% |
|  | | | | | |
| Tonsil | vCJD | Frozen | 33 | 26.3 (8.0) | 42.4% |
| (Total 193) |  | Formalin-Fixed | 32* | 25.9 (7.8) | 43.8% |
|  |  | FFPE | - | - | - |
|  | sCJD | Frozen | 7 | 46.7 (13.9) | 28.6% |
|  |  | Formalin-Fixed | 7* | 46.7 (13.9) | 28.6% |
|  |  | FFPE | - | - | - |
|  | Control | Frozen | 38 | 13.0 (12.6) | 39.5% |
|  |  | Formalin-Fixed | 38* | 13.0 (12.6) | 39.5% |
|  |  | FFPE | 38* | 13.0 (12.6) | 39.5% |
| *Test Dataset* |  |  |  |  |  |
| Appendix  (Total 96) | vCJD | Frozen | 8 | 32.8 (13.1) | 25.0% |
|  |  | Formalin-Fixed | 8* | 32.8 (13.1) | 25.0% |
|  | sCJD | Frozen | 12 | 65.0 (13.7) | 50.0% |
|  |  | Formalin-Fixed | 12* | 65.0 (13.7) | 50.0% |
|  | PrP-positive | FFPE | 20 | 30.1 (16.0) | 45.0% |
|  | PrP-negative (Control) | FFPE | 36 | 33.7 (11.7) | 52.8% |

Supplementary Table 1. Characteristics of the samples used in the study**.** *Different fixation status samples are duplicates of those in the adjacent row. Where sample numbers in adjacent rows are unequal, the lower number is a subset of the larger number.

| QC criterion | # of samples removed (*Training dataset*) | # of samples removed (*Test dataset*) |
| --- | --- | --- |
| Abnormal beta distribution / failed CpG fraction > 0.1 | 24 | 18 |
| Sex mismatch | 3 | 0 |
|  | | |
|  | **# of CpGs removed (*Training dataset*)** | **# of CpGs removed (*Test dataset*)** |
| Detection P-value above 0.01 | 167,042 | 173,553 |
| No CG start | 1612 | 1709 |
| Probes with SNPs | 80,891 | 81,576 |
| MultiHit | 14 | 18 |
| XY chromosome | 10,031 | 9965 |
|  | | |
| Remainder | 324 samples 606,501 CpGs | 78 samples 599,417 CpGs |
| Merged dataset | 402 samples 557,431 CpGs |  |

Supplementary Table 2. Description of array processing and quality control of the data for further analyses**.** To filter low quality samples, a visual inspection of the beta distribution, an analysis of the fraction of CpG failed and sex matching were done. To filter low quality CpGs, the ChAMP package filter() function was used, which removes (1) probes with detection p-value over threshold of 0.01, (2) non-CpG probes, (3) SNP-related probes from Zhou et al. 2017(19), (4) multi-hit probes from Nordlund et al. 2013(20), and (5) probes located in chromosome X and Y.

| Tissue | Diagnosis | Group | Mean r – Pairs (n) | | Mean r – NonPairs (n) | Mean r – Fr (n) | Mean r – FF (n) | Mean r – FFPE (n) |
| --- | --- | --- | --- | --- | --- | --- | --- | --- |
| Appendix | vCJD | Fr-FF | 0.9649 ±0.0142 (6) | | 0.9559 ±0.0140 (54) | 0.9759 ±0.0129 (45) | 0.9535 ±0.0166 (15) | NA |
|  | sCJD | Fr-FF | 0.9802 ±0.0055 (37) | | 0.9719 ±0.0149 (1369) | 0.9771 ±0.0131 (666) | 0.9723 ±0.0153 (703) | NA |
|  | Control | NA | NA | | NA | NA | NA | 0.9734 ±0.0091 (1225) |
|  | | | |  |  |  |  |  |
| Tonsil | vCJD | Fr-FF | 0.9817 ±0.0105 (28) | | 0.9758 ±0.0128 (900) | 0.9878 ±0.0108 (496) | 0.9642 ±0.0152 (406) | NA |
|  | sCJD | Fr-FF | 0.9784 ±0.0234 (7) | | 0.9598 ±0.0369 (42) | 0.9819 ±0.0150 (21) | 0.9483 ±0.0404 (21) | NA |
|  | Control | Fr-FF | 0.9774 ±0.0159 (32) | | 0.9721 ±0.0159 (1184) | 0.9914 ±0.0032 (703) | 0.9534 ±0.0217 (496) | 0.9832 ±0.0132 (703) |
|  |  | Fr-FFPE | 0.9922 ±0.0090 (38) | | 0.9868 ±0.0094 (1406) | NA | NA | NA |
|  |  | FF-FFPE | 0.9735 ±0.0176 (32) | | 0.9676 ±0.0181 (1184) | NA | NA | NA |

Supplementary Table 3. Correlation between paired frozen (Fr), formalin-fixed (FF) and FFPE samples, compared with unpaired samples and background correlation in each diagnosis group**.** Correlations between paired samples (ie those derived from the same original sample but processed differently) are stronger that the correlations between unpaired samples in all groups. However correlations between paired samples are not stronger compared to the background correlation levels between frozen samples. Background correlation levels of formalin-fixed samples is generally lower compared to background correlation levels of frozen and FFPE samples.

**References**

1. Prusiner SB. Novel proteinaceous infectious particles cause scrapie. Science. 1982;216(4542):136-44.

2. Ironside JW, Hilton DA, Ghani A, Johnston NJ, Conyers L, McCardle LM, et al. Retrospective study of prion-protein accumulation in tonsil and appendix tissues. Lancet. 2000;355(9216):1693-4.

3. Gill ON, Spencer Y, Richard-Loendt A, Kelly C, Dabaghian R, Boyes L, et al. Prevalent abnormal prion protein in human appendixes after bovine spongiform encephalopathy epizootic: large scale survey. BMJ. 2013;347:f5675.

4. Gill ON, Spencer Y, Richard-Loendt A, Kelly C, Brown D, Sinka K, et al. Prevalence in Britain of abnormal prion protein in human appendices before and after exposure to the cattle BSE epizootic. Acta Neuropathol. 2020;139(6):965-76.

5. Frosh A, Smith LC, Jackson CJ, Linehan JM, Brandner S, Wadsworth JD, et al. Analysis of 2000 consecutive UK tonsillectomy specimens for disease-related prion protein. Lancet. 2004;364(9441):1260-2.

6. Hilton DA, Ghani AC, Conyers L, Edwards P, McCardle L, Ritchie D, et al. Prevalence of lymphoreticular prion protein accumulation in UK tissue samples. J Pathol. 2004;203(3):733-9.

7. Dabin LC, Guntoro F, Campbell T, Bélicard T, Smith AR, Smith RG, et al. Altered DNA methylation profiles in blood from patients with sporadic Creutzfeldt–Jakob disease. 2020;140(6):863-79.

8. Fortin JP, Triche TJ Jr, Hansen KD. Preprocessing, normalization and integration of the Illumina HumanMethylationEPIC array with minfi. Bioinformatics. 2017 Feb 15;33(4):558-560.

9. Tian Y, Morris TJ, Webster AP, Yang Z, Beck S, Feber A, Teschendorff AE. ChAMP: updated methylation analysis pipeline for Illumina BeadChips. Bioinformatics. 2017 Dec 15;33(24):3982-3984.

10. Ritchie ME, Phipson B, Wu D, Hu Y, Law CW, Shi W, Smyth GK. limma powers differential expression analyses for RNA-sequencing and microarray studies. Nucleic Acids Res. 2015 Apr 20;43(7):e47.

11. Gjessing HK and Lie RT. Case-parent triads: Estimating single- and double-dose effects of fetal and maternal disease gene haplotypes. Annals of Human Genetics (2006) 70, pp. 382-396.

12. Huang da W, Sherman BT, Lempicki RA. Bioinformatics enrichment tools: paths toward the comprehensive functional analysis of large gene lists. Nucleic Acids Res. 2009;37(1):1-13.

13. Huang da W, Sherman BT, Lempicki RA. Systematic and integrative analysis of large gene lists using DAVID bioinformatics resources. Nat Protoc. 2009;4(1):44-57.

14. Krijthe JH (2015). Rtsne: T-Distributed Stochastic Neighbor Embedding using Barnes-Hut Implementation. R package version 0.16.15. Capper D, Jones DTW, Sill M, Hovestadt V, Schrimpf D, Sturm D, et al. DNA methylation-based classification of central nervous system tumours. Nature. 2018;555(7697):469-74.

16. A. Liaw and M. Wiener (2002). Classification and Regression by randomForest. R News 2(3), 18--22.

17. Moran S, Vizoso M, Martinez-Cardus A, Gomez A, Matias-Guiu X, Chiavenna SM, et al. Validation of DNA methylation profiling in formalin-fixed paraffin-embedded samples using the Infinium HumanMethylation450 Microarray. Epigenetics. 2014;9(6):829-33.

18. de Ruijter TC, de Hoon JP, Slaats J, de Vries B, Janssen MJ, van Wezel T, et al. Formalin-fixed, paraffin-embedded (FFPE) tissue epigenomics using Infinium HumanMethylation450 BeadChip assays. Lab Invest. 2015;95(7):833-42.

19. Zhou W, Laird PW, Shen H. Comprehensive characterization, annotation and innovative use of Infinium DNA methylation BeadChip probes. Nucleic Acids Res. 2017;45(4):e22.

20. Nordlund J, Backlin CL, Wahlberg P, Busche S, Berglund EC, Eloranta ML, et al. Genome-wide signatures of differential DNA methylation in pediatric acute lymphoblastic leukemia. Genome Biol. 2013;14(9):r105.
